# The right patient in the right place: the central role of infection control

**DOI:** 10.1186/2047-2994-4-S1-P268

**Published:** 2015-06-16

**Authors:** V Pinto, A Bispo, C Palos, L Caldas

**Affiliations:** 1 Infection Control and Antibiotics Committee, Hospital Beatriz Ângelo, Loures, Portugal; 2 Risk Management Committee, Hospital Beatriz Ângelo, Loures, Portugal

## Introduction

The Portuguese reality of high prevalence of Epidemiologically Important Microrganisms (EIM) combined with the shortage of single rooms led to the implementation of a real-time action strategy, in order to achieve best practices of isolation, starting on admission.

## Objectives

Optimization of site management of patients, centered on the Infection Control and Antibiotics Committee (ICAC) on a paper-free hospital.

## Methods

ICAC implemented a methodology based on the relationship between the Electronic Medical Registry (EMR), Lab, Infection Control Nurse (ICN) and the Site Manager Nurse (SMN), comprising the following steps: 1)At inpatient admission, namely on the Emergency Room (ER), a 8 question tool named Electronic Epidemiological Query on Admission (EEQA) is fulfield by the physician in charge of the patient, generating automated micro prescriptions and isolation procedures; 2)ICN daily (monday to friday) receives data from the micro lab and alerts from hospital information system. Based on the analysis of this information, each patient is studied and the need of isolation is assessed. ICN works along SMN and nurses and physicians on the wards, deciding their allocation into single rooms or into cohorts; 3)An Infection Control Note is written on the patient EMR, which migrates automatically to its Discharge Note; 4)SMN acts according to data from the EEQA, the ICN and also from physicians and nurses in charge of patients; 5)Finally, a phone contact to the staff on duty is made in order to transmit the right isolation needs and an email is sent to the respective Medical Director and Head Nurse.

## Results

According to this strategy, in last December an average of 3.29 daily changes (placement on individual rooms, negative pressure rooms or cohorting) were made in patient´s allocation on a total of 3274 admissions.

## Conclusion

Based on this model, ICAC ensures that all patients admitted were properly allocated, according to information obtained from the Electronic Epidemiological Query on Admission and EIM detected. Infection Control Nurse has a central role along with Site Manager Nurse, thus reducing the risk of cross-transmission and contributing to Patient Safety on a context of shortage of single rooms.

## Disclosure of interest

None declared.

